# Exploring Extravasation in Cancer Patients

**DOI:** 10.3390/cancers16132308

**Published:** 2024-06-24

**Authors:** Tuan D. Pham, Taichiro Tsunoyama

**Affiliations:** 1 Barts and The London School of Medicine and Dentistry, Queen Mary University of London, London E1 2AD, UK; 2 School of Medicine, Teikyo University, Tokyo 173-0003, Japan; taichirotsunoyama@yahoo.co.jp

**Keywords:** extravasation, cancer treatment, chemotherapy, radiotherapy, prevention and management, emerging technologies

## Abstract

**Simple Summary:**

This research explores the accidental leakage of substances from veins during cancer treatments like chemotherapy and radiotherapy, a problem known as extravasation. The study aims to understand the causes, risks, symptoms, and long-term effects of extravasation and to find better ways to prevent and manage it. New technologies and methods, such as advanced imaging and smart catheters, are highlighted as potential solutions. By identifying gaps in the current knowledge and suggesting future research areas, this study aims to improve patient safety and treatment outcomes, offering valuable insights for healthcare professionals and emphasizing the need for continued innovation in cancer care.

**Abstract:**

Extravasation, the unintended leakage of intravenously administered substances, poses significant challenges in cancer treatment, particularly during chemotherapy and radiotherapy. This comprehensive review explores the pathophysiology, incidence, risk factors, clinical presentation, diagnosis, prevention strategies, management approaches, complications, and long-term effects of extravasation in cancer patients. It also outlines future directions and research opportunities, including identifying gaps in the current knowledge and proposing areas for further investigation in extravasation prevention and management. Emerging technologies and therapies with the potential to improve extravasation prevention and management in both chemotherapy and radiotherapy are highlighted. Such innovations include advanced vein visualization technologies, smart catheters, targeted drug delivery systems, novel topical treatments, and artificial intelligence-based image analysis. By addressing these aspects, this review not only provides healthcare professionals with insights to enhance patient safety and optimize clinical practice but also underscores the importance of ongoing research and innovation in improving outcomes for cancer patients experiencing extravasation events.

## 1. Introduction

Extravasation, in the context of cancer treatment, refers to the unintended leakage of chemotherapy drugs, embolic agents, or other intravenously administered substances from the blood vessel into surrounding tissues [1]. This phenomenon can occur during the infusion process, causing tissue damage, pain, and other complications. In severe cases, extravasation can lead to compartment syndrome, which is a serious and potentially life-threatening condition resulting from pressure buildup due to internal bleeding or tissue swelling.

**Figure 1:**
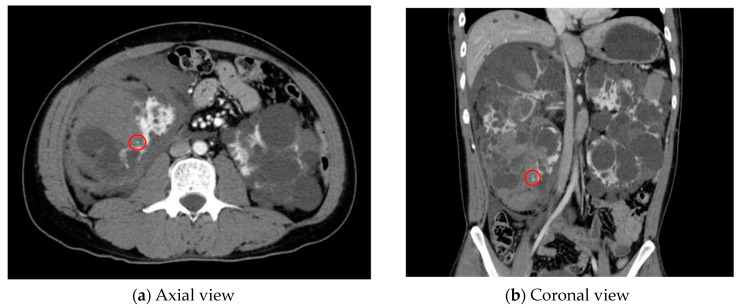
Extravasation-induced hemorrhage shown in (**a**,**b**) with circles from a polycystic kidney.

**Figure 2:**
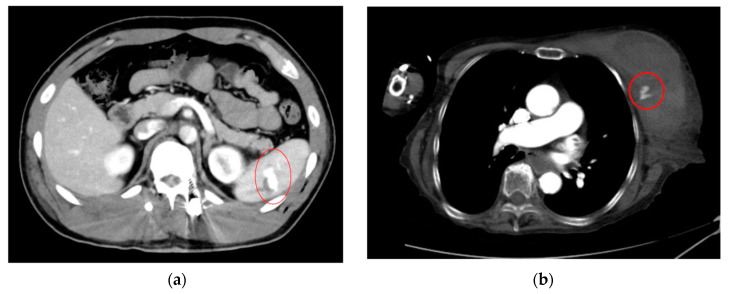
Extravasation-induced hemorrhage shown with circles: pseudoaneurysm after spleen injury (**a**), and (**b**) muscle hematoma in chest.

**Figure 3:**
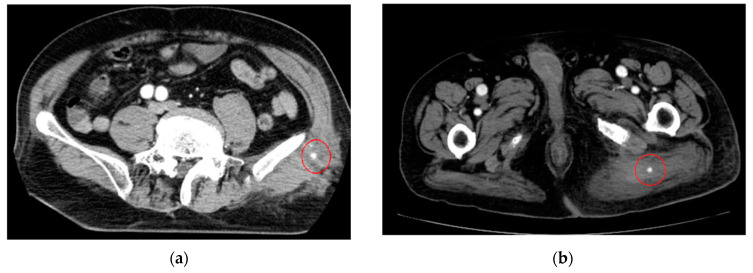
Extravasation-induced hemorrhage marked with circles on fractured pelvic (**a**) and intragluteal hematoma (**b**).

As an example, Figure 1 shows extravasation-induced hemorrhage from a polycystic kidney. Identifying the precise bleeding site on the computed tomography (CT) image presented a challenge. However, upon reviewing the angiography findings, it became evident that the bleeding originated from the area depicted in the CT image. In Figure 2, two instances of extravasation-induced hemorrhage are depicted, marked with the circles. Firstly, (a) displays a pseudoaneurysm visible post-spleen injury. Secondly, (b) illustrates a muscle hematoma in the chest region. In Figure 3, two cases of extravasation-induced hemorrhage are evident. In (a), circles mark extravasation on a fractured pelvic region, indicating a significant injury. Meanwhile, (b) showcases an intragluteal hematoma, highlighting the varied presentations of extravasation across different anatomical contexts. These CT images provide valuable insights into the varied presentations of extravasation cases across different anatomical contexts.

Chemotherapy agents are powerful medications used to treat cancer by targeting rapidly dividing cells. However, many of these drugs are vesicants, meaning they can cause severe tissue damage if they leak outside the blood vessel [2,3]. Extravasation can occur due to various factors [4], including poorly functioning intravenous (IV) catheters, improper drug administration techniques, or fragile blood vessels in cancer patients [5,6].

In certain carefully selected situations, a contrast medium may be used during the virtual simulation phase of radiotherapy planning. This phase involves imaging studies such as CT or MRI scans, which are essential for accurately mapping the tumor and surrounding tissues. Injecting a contrast medium during this phase enhances the visibility of internal structures, allowing for precise targeting of the radiation beams.

However, the use of a contrast medium is not without risks. One potential issue is extravasation, which is the accidental leakage of contrast medium from blood vessels into surrounding tissues. This can occur during the injection process and may cause discomfort and local tissue damage [7,8]. Fortunately, extravasation is generally managed promptly and effectively, minimizing any disruption to the overall planning process [9].

Once the treatment plan is established during the virtual simulation phase, the actual delivery of radiotherapy does not involve the use of contrast media. During treatment sessions, the radiation beams are directed according to the pre-determined plan based on the detailed images obtained with contrast during the planning phase. No additional contrast medium is needed or administered during the radiation delivery itself.

This protocol is followed to avoid any risks or complications associated with the use of a contrast medium during the actual delivery of radiation. The planning scans with contrast provide the necessary anatomical detail for accurate treatment, and the execution of the treatment plan proceeds without the need for additional contrast, ensuring a safer and more controlled environment for the patient. This approach highlights the careful and precise nature of radiotherapy planning and delivery, emphasizing the importance of each phase in achieving optimal treatment outcomes.

In summary, while the use of a contrast medium during the virtual simulation phase of radiotherapy planning can significantly enhance the accuracy of tumor mapping and radiation targeting, it is only used during the planning phase. The treatment phase itself relies on the detailed images obtained earlier, avoiding further use of a contrast medium and its associated risks. This careful and methodical approach ensures that patients receive the most effective and safe radiotherapy treatment possible.

The consequences of extravasation, both in chemotherapy and radiotherapy, can vary depending on factors such as the type and amount of drug involved, the location of the extravasation, and the promptness of intervention [2,10]. Mild cases may cause local discomfort, erythema, and swelling, while severe cases can lead to tissue necrosis, ulceration, and long-term functional impairment. In radiotherapy, extravasation events can result in similar local tissue damage and complications, depending on the type and dose of radiation delivered and the duration of exposure.

Extravasation not only poses immediate risks to patients’ health and well-being but can also impact the effectiveness of cancer treatment and compromise future treatment options [11]. Therefore, prevention, early detection, and prompt management of extravasation events are crucial components of comprehensive cancer care.

Healthcare providers involved in cancer treatment must be vigilant in assessing patients for signs of extravasation during and after chemotherapy infusion [4,12]. Timely intervention, which may include stopping the infusion, applying appropriate antidotes or treatments, and providing supportive care, can help minimize tissue damage and mitigate the impact of extravasation on patients’ outcomes. Understanding extravasation in the context of cancer treatment is essential for healthcare professionals to optimize patient safety, ensure the effectiveness of cancer therapy, and provide comprehensive, supportive care to individuals undergoing treatment for cancer.

**Figure 4:**
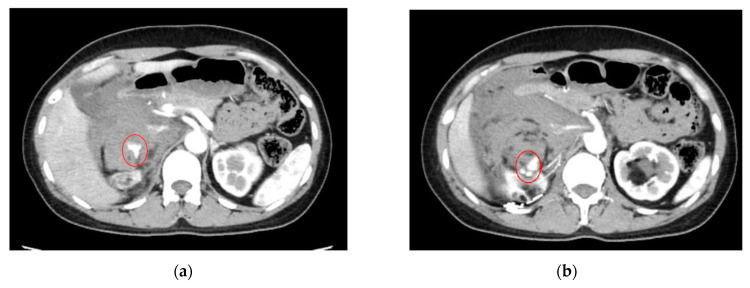
Extravasation-indued hemorrhage from kidney angiomyolipoma for both (**a**,**b**) shown with circles.

As an illustration, Figure 4 depicts an extravasation-induced hemorrhage from kidney angiomyolipoma, a condition characterized by the formation of benign tumors in the kidney. Patients with this condition may experience a range of symptoms, including anemia, fever, pain, or high blood pressure. In cases where tumors grow, treatment such as embolization or surgery may be necessary to mitigate the risk of bleeding.

**Figure 5:**
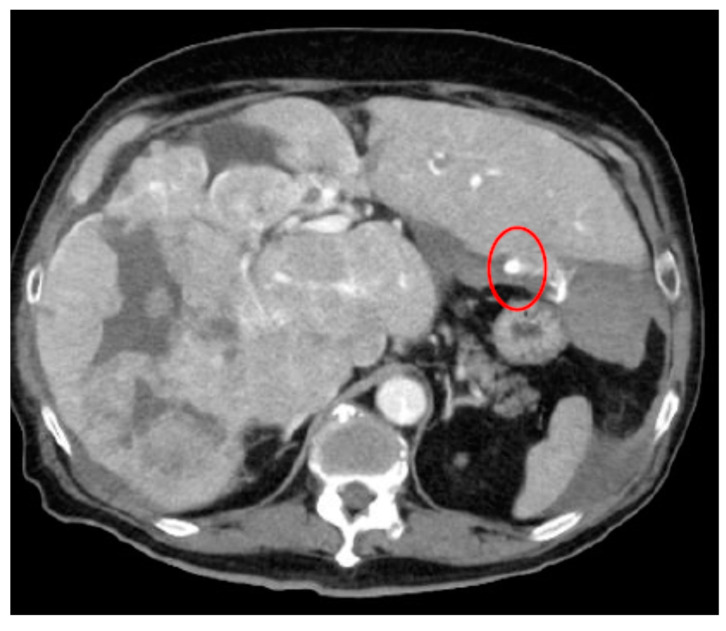
Extravasation-induced hemorrhage marked with a circle due to the rupture of hepatocellular carcinoma.

Figure 5 illustrates an extravasation-induced hemorrhage resulting from the rupture of hepatocellular carcinoma in a 68-year-old male patient. This individual had previously undergone transcatheter arterial chemoembolization, a minimally invasive and targeted treatment utilized for managing certain advanced liver tumors that are not amenable to surgical removal. Following the rupture, the patient was admitted to the hospital in a state of hypotension, characterized by low blood pressure. This case underscores the importance of closely monitoring patients with hepatocellular carcinoma, particularly those who have undergone chemoembolization, for potential complications such as extravasation.

**Figure 6:**
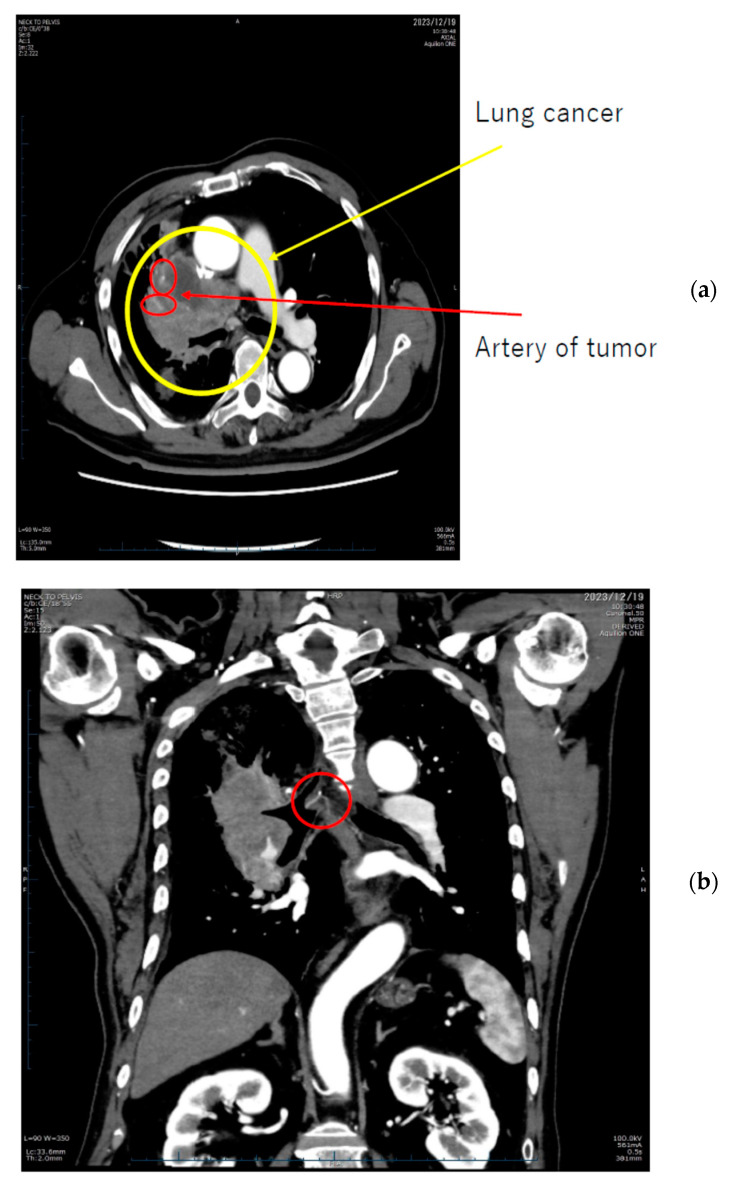
Lung cancer extravasation sites highlighted with circles in (**a**,**b**).

Additionally, Figure 6 delineates the locations of extravasation detected in a patient’s lung cancer as visualized on CT images. Specifically, in panel (b), one of the feeding arteries of the tumor (the right bronchial arterial) is depicted. Identifying the responsible vessel on CT proved challenging, leading to the decision to embolize this artery. The patient had previously experienced hemoptysis prior to initiating chemotherapy.

## 2. Extravasation and Its Significance in Cancer Care

The implications of extravasation extend beyond immediate physical discomfort, encompassing both chemotherapy and radiotherapy. Extravasation events can impact the effectiveness of cancer treatment by reducing the delivery of chemotherapy or radiation to the intended target site. This can compromise the therapeutic efficacy of the treatment regimen and potentially affect treatment outcomes [13]. Furthermore, severe extravasation injuries may necessitate treatment interruptions or modifications, leading to delays in cancer therapy and potentially limiting future treatment options [14].

In addition to the direct physical effects, extravasation can also have psychological implications for patients undergoing cancer treatment [15]. The experience of pain, disfigurement, or long-term complications resulting from extravasation can contribute to increased anxiety, distress, and decreased quality of life for the affected individuals.

Given its potential to cause significant harm and disrupt cancer treatment, extravasation prevention, early detection, and prompt management are critical components of comprehensive cancer care [2,16]. Healthcare providers must be vigilant in assessing patients for signs of extravasation during chemotherapy or radiotherapy infusion, and prompt intervention is essential to minimize tissue damage and mitigate the impact on patients’ outcomes [2,4,12]. Strategies for preventing extravasation include proper vascular access device selection and placement, computerized detection methods, meticulous administration techniques, and patient education on recognizing and reporting signs of extravasation.

## 3. Review Scope and Objectives

The scope of this review covers an in-depth exploration of extravasation in patients undergoing cancer treatment, encompassing both chemotherapy-induced and radiotherapy-associated extravasation events. The review aims to provide a comprehensive understanding of extravasation, including its pathophysiology, incidence, risk factors, clinical presentation, diagnosis, prevention strategies, management approaches, complications, and long-term effects.

The objectives of this review include the following aspects.

- Elucidating the pathophysiology of extravasation: This includes examining the mechanisms by which chemotherapy agents and radiotherapy cause tissue damage upon extravasation and identifying factors that influence the severity of extravasation reactions.
- Evaluating the incidence and risk factors associated with extravasation: This involves analyzing the available data on the prevalence of extravasation in cancer patients and identifying patient-related and treatment-related factors that predispose individuals to extravasation events.
- Clinical presentation and diagnosis of extravasation: This includes describing the typical signs and symptoms of extravasation, as well as reviewing the diagnostic tools and techniques used in confirming extravasation.
- Reviewing preventive strategies for extravasation: This part outlines the measures aimed at reducing the risk of extravasation during chemotherapy and radiotherapy administration, such as proper vascular access device selection, administration techniques, and patient education.
- Exploring management approaches for extravasation events: This involves presenting current guidelines and protocols for managing extravasation injuries, including pharmacological and non-pharmacological interventions.
- Complications and long-term effects of extravasation: This includes exploring potential complications arising from extravasation, as well as the impact of extravasation on patients’ quality of life and treatment outcomes.
- Patient education and support: This involves highlighting the importance of patient education regarding extravasation risks and early symptom recognition, as well as strategies for providing psychological support to the affected individuals.
- Identifying future research directions and opportunities: This includes identifying gaps in the current knowledge, areas for further investigation, and emerging technologies or therapies that may improve extravasation prevention and management.

By addressing these objectives, this review aims to provide healthcare professionals and researchers with a comprehensive resource for understanding, preventing, and managing extravasation in patients undergoing cancer treatment, including both chemotherapy and radiotherapy, ultimately contributing to enhanced patient safety and improved clinical outcomes.

## 4. Pathophysiology of Extravasation

Both chemotherapy and radiotherapy can induce extravasation through complex mechanisms involving direct tissue toxicity, vascular damage, altered tissue perfusion, and secondary effects from tumor response. Understanding these mechanisms is crucial for the effective management of extravasation events in cancer treatment, thereby minimizing patient morbidity and optimizing treatment outcomes.

### 4.1. Extravasation in Chemotherapy

Many chemotherapy drugs are highly potent cytotoxic agents designed to target rapidly dividing cancer cells. However, these drugs can also be highly toxic to healthy tissues if they inadvertently leak into the surrounding extracellular space. Chemotherapy agents can induce chemical irritation and tissue toxicity upon extravasation, leading to local inflammation, pain, and tissue necrosis. The severity of tissue damage depends on factors such as the concentration and potency of the drug, the volume extravasated, and the duration of exposure [2,17].

Chemotherapy agents can directly damage endothelial cells lining the blood vessels, leading to disruption of vascular integrity [18,19]. This damage can manifest as endothelial cell death, increased vascular permeability, and impaired endothelial function. 

Some chemotherapy drugs, particularly those administered in high concentrations or volumes, can exert mechanical pressure on the blood vessels, leading to vascular compression and compromised blood flow [20]. This vascular compression can induce localized ischemia, further exacerbating tissue damage and increasing the risk of extravasation [21]. Additionally, ischemic tissues may be more susceptible to the toxic effects of chemotherapy agents, amplifying the severity of extravasation injuries and increasing cardiovascular risk in cancer patients [22]. 

Extravasation events can occur due to various vascular access-related issues, including catheter displacement, infiltration, or leakage around the insertion site [23]. The improper placement, maintenance, or malfunction of vascular access devices, such as central venous catheters or peripherally inserted central catheters, can increase the risk of chemotherapy leakage into surrounding tissues. Additionally, patient factors such as poor venous access, vascular fragility, or obesity may further predispose individuals to extravasation events [24,25].

### 4.2. Extravasation in Radiotherapy

Radiotherapy delivers ionizing radiation precisely to tumor tissues while minimizing exposure to surrounding healthy tissues. However, unintended radiation exposure to adjacent blood vessels can cause direct damage to the endothelial cells lining the vessel walls [26,27]. Radiation-induced endothelial cell death, inflammation, and fibrosis can weaken blood vessel integrity and increase permeability, facilitating the extravasation of blood or fluid into surrounding tissues [28].

Radiation therapy can induce secondary effects on vascular integrity through inflammatory responses and fibrotic changes in the irradiated tissues [29]. Chronic inflammation and fibrosis can lead to progressive vascular remodeling and sclerosis [30] and increase the risk of extravasation [31]. Additionally, radiation-induced endothelial dysfunction and thrombosis can further exacerbate vascular damage, predisposing vessels to leakage and extravasation events [28,32].

Radiation therapy can induce complex alterations in tissue perfusion and microvascular architecture within the irradiated field. Radiation-induced microvascular damage, capillary rarefaction, and thrombotic occlusion can disrupt tissue perfusion dynamics, leading to localized ischemia and hypoxia [33]. These microvascular changes can compromise vascular integrity and increase the susceptibility of blood vessels to extravasation, particularly in tissues with pre-existing vascular compromise or impaired healing capacity [34].

The tumor’s response to radiation therapy, such as tumor shrinkage, necrosis, or vascular regression [35,36], can indirectly contribute to extravasation events. Radiation-induced tumor cell death and vascular changes can alter the structural integrity and perfusion dynamics of the tumor microenvironment, increasing the risk of vascular leakage and extravasation. Additionally, radiation-induced tumor regression may lead to changes in tissue architecture and vascular distribution [37], further predisposing vessels to extravasation injuries.

Understanding extravasation during radiotherapy and its potential effects on the microenvironment is complex. It requires a contextual grasp of different radiotherapy fractionation schedules and treatment modalities. Examining the impact of radiation on tumor and vascular structures highlights the interplay between radiation-induced changes and the risk of extravasation, emphasizing the need for tailored treatment approaches to mitigate such risks.

Fractionation refers to dividing the total dose of radiation into multiple smaller doses delivered over several sessions [38,39]. This approach is standard in conventional radiotherapy and has several key advantages, including minimizing the damage to healthy tissues and allowing for cellular repair between sessions. Traditional fractionation typically involves daily treatments over several weeks, which reduces the likelihood of significant changes in vascular permeability and subsequent extravasation.

Different fractionation schedules can have varying biological effects [40]. Hypofractionation [41], which involves delivering larger doses in fewer sessions, and hyperfractionation [42], which delivers smaller doses multiple times a day, each having a unique impact on the tissue response and tumor microenvironment. Understanding these distinctions is essential when evaluating the risk of extravasation and the resultant microenvironmental changes.

Stereotactic radiotherapy (SRT) [43] and stereotactic body radiotherapy (SBRT) [44] are specialized forms of radiotherapy that deliver high doses of radiation in a few fractions with extreme precision. These treatments are typically used for small, well-defined targets and can induce significant biological effects due to the high dose per fraction. Research has shown that high-dose radiation can increase vascular permeability and potentially lead to extravasation, altering the local microenvironment. These changes can influence the tumor’s behavior, immune response, and treatment outcomes. The effects observed in SRT and SBRT differ from those in conventional fractionation due to the differences in dose intensity and distribution.

Integrating these considerations provides insights into extravasation in radiotherapy, highlighting how different fractionation schedules and treatment modalities can influence the risk and impact of extravasation. This approach underscores the importance of tailoring radiotherapy strategies to individual patient and tumor characteristics to optimize outcomes and minimize adverse effects.

To obtain a comprehensive understanding, it is crucial to consider the radiobiological principles that govern the tissue response to radiation [45,46]. Key concepts include the four Rs of radiobiology (repair, redistribution, repopulation, and reoxygenation) [47] and the linear-quadratic model [48], which describes cell survival in response to varying doses of radiation. These principles help explain why different fractionation schedules result in varied biological outcomes.

By applying these radiobiological principles, healthcare providers can better predict and manage the risks associated with extravasation. This knowledge enables the development of more effective and safer radiotherapy plans, ensuring that treatments are both precise and tailored to the needs of each patient. This holistic approach aims to maximize the therapeutic benefits while minimizing potential adverse effects, leading to improved patient outcomes.

### 4.3. Mechanisms of Tissue Damage Induced by Chemotherapy and Radiation Extravasation

Extravasation of chemotherapy drugs and radiation therapy can cause tissue damage through multiple mechanisms, including chemical toxicity, vascular injury, ischemic injury, direct cellular damage, inflammatory responses, and vascular fibrosis. Understanding these mechanisms is crucial for implementing preventive measures, early detection, and prompt management of extravasation injuries to minimize patient morbidity and optimize the treatment outcomes in cancer care.

Chemotherapy-induced tissue damage encompasses a spectrum of factors that contribute to adverse effects upon extravasation. Chemotherapy agents, known for their high cytotoxicity, exert direct harm on cells and tissues upon inadvertent leakage [49]. These agents induce chemical irritation and toxic effects on the surrounding tissues, eliciting inflammatory responses characterized by pain and tissue necrosis. Furthermore, chemotherapy drugs disrupt the integrity of blood vessel walls, resulting in endothelial cell damage and heightened vascular permeability [18]. This vascular injury compromises normal vascular function, facilitating the extravasation of chemotherapy agents into the surrounding tissues [19]. Additionally, certain chemotherapy drugs can induce local vasoconstriction or impair tissue perfusion, leading to tissue ischemia and hypoxia. The resultant ischemic injury exacerbates tissue damage and impedes wound healing processes, amplifying the severity of extravasation injuries [21].

On the other hand, radiation-induced tissue damage arises from various factors intrinsic to radiotherapy. Ionizing radiation, utilized in radiotherapy, directly inflicts damage on cells and tissues by inducing DNA damage and oxidative stress [50]. When blood vessels are exposed to radiation, endothelial cells undergo apoptosis, culminating in vascular damage and increased permeability. Concurrently, radiation therapy prompts an inflammatory response within irradiated tissues, prompting the release of pro-inflammatory cytokines and the recruitment of immune cells. Prolonged inflammation exacerbates tissue damage and hampers wound healing, thereby prolonging the effects of extravasation [51]. Moreover, radiation-induced fibrosis of blood vessels and surrounding tissues can occur due to the activation of fibroblasts and deposition of extracellular matrix proteins. This vascular fibrosis compromises vascular function, exacerbating tissue ischemia and elevating the risk of necrosis and impaired tissue repair [52].

In cases where patients undergo both chemotherapy and radiation therapy concurrently or sequentially, the combined effects of chemotherapy and radiation extravasation can synergistically exacerbate tissue injury. Extravasation of chemotherapy drugs and radiation-induced tissue damage mutually potentiate each other, resulting in more severe and prolonged effects. Furthermore, compromised healing processes stemming from chemotherapy-induced immunosuppression and radiation-induced tissue fibrosis impede the normal wound healing cascade following extravasation injuries. Delayed or impaired wound healing heightens the risk of infection, scarring, and long-term functional impairment, underscoring the importance of timely intervention and comprehensive management strategies in mitigating the adverse consequences of extravasation in cancer care [53].

### 4.4. Factors Influencing the Severity of Extravasation Reactions in Both Treatment Modalities

The severity of extravasation reactions in chemotherapy and radiotherapy is influenced not only by patient characteristics and treatment variables but also by a range of environmental and systemic factors that interact dynamically throughout the treatment process [5].

Patient-related factors encompass a spectrum of considerations that impact extravasation reactions. Patients with pre-existing conditions affecting vascular integrity, such as diabetes or peripheral vascular disease, may be at heightened risk of severe reactions due to compromised tissue perfusion and healing capacity [54,55]. Additionally, factors such as smoking history, nutritional status, and immune function can influence tissue resilience and susceptibility to injury, further complicating the assessment and management of extravasation events [56].

Treatment-related factors play an important role in shaping the severity and trajectory of extravasation reactions. The choice of chemotherapy regimen, including the specific agents used and their concentration, formulation, and infusion rate, can significantly impact tissue tolerance and susceptibility to injury. Certain chemotherapy drugs, such as anthracyclines or vinca alkaloids, are notorious for their vesicant properties and high potential for tissue damage if extravasated [2]. Similarly, in radiotherapy, factors such as the total radiation dose, fractionation schedule, and beam energy distribution can influence the depth of tissue penetration and the extent of radiation-induced damage, with higher doses and prolonged treatment courses increasing the risk of severe reactions [13].

Environmental and procedural factors also contribute to the complexity of extravasation management [11,12,53]. The type and placement of vascular access devices, such as central venous catheters or peripherally inserted central catheters, can influence the ease of drug administration and the likelihood of extravasation. Moreover, the skill and experience of healthcare providers in catheter insertion, maintenance, and monitoring play a critical role in preventing and mitigating extravasation events. Adequate patient education and counseling regarding the signs and symptoms of extravasation, as well as the importance of early reporting and intervention, are essential for empowering patients to actively participate in their care and minimize the risk of complications.

The severity of extravasation reactions in chemotherapy and radiotherapy is influenced by interconnected factors spanning patient characteristics, treatment variables, and environmental considerations. A comprehensive understanding of these factors is essential for healthcare professionals to anticipate, recognize, and effectively manage extravasation events, thereby minimizing patient morbidity and optimizing treatment outcomes in cancer care. By adopting a multidisciplinary approach that addresses the complex interplay of patient, treatment, and environmental factors, healthcare teams can enhance patient safety and quality of care throughout the cancer treatment journey.

## 5. Incidence and Risk Factors

While precise incidence rates vary, existing data offer valuable insights into the frequency of extravasation events in cancer patients undergoing chemotherapy and radiotherapy.

For chemotherapy, past studies indicated that the overall incidence of extravasation ranges from 0.1% to 6% [57]. Another study found that the occurrence rates of chemotherapy extravasation varied significantly, with estimates ranging from as low as 0.01% to as high as 7% [4]. This variability can be attributed to factors such as the specific chemotherapy regimen used, the route of administration (IV, intramuscular, or subcutaneous), and the type of vascular access device employed. Certain chemotherapy agents, notably anthracyclines like doxorubicin and vinca alkaloids such as vincristine, are more commonly associated with extravasation due to their vesicant or irritant properties. 

In contrast, the incidence of extravasation during radiotherapy is relatively rare, typically falling below 1% [11]. The occurrence rate of extravasation among cancer patients undergoing CT is relatively high, with reported incidences ranging from 0.25% to 1.2% [58], influenced by factors such as the type and location of radiation treatment, as well as patient-specific characteristics. External beam radiation therapy is generally associated with a lower risk of extravasation compared to internal radiation techniques like brachytherapy. However, extravasation incidents can still occur, particularly in areas where radiation fields intersect with major blood vessels or superficial tissues.

The administration of IV contrast plays a significant role in diagnostic radiology, yet it is not devoid of risks. These risks include local and systemic allergic reactions, as well as the potential for subcutaneous extravasation of contrast media. While subcutaneous extravasation of contrast medium is a relatively uncommon complication, it is widely acknowledged. Fortunately, the majority of incidents are minor and can be addressed through conservative management. However, there are rare cases that necessitate immediate surgical intervention [59].

While extravasation is infrequent, its occurrence can have significant implications for patient safety and treatment outcomes. Prompt recognition and management are crucial in minimizing tissue damage and optimizing patient care. Healthcare providers must remain vigilant in monitoring patients for signs and symptoms of extravasation, implementing preventive measures, and promptly intervening when an extravasation event is suspected.

## 6. Clinical Presentation and Diagnosis

### 6.1. Typical Signs and Symptoms of Extravasation in Patients Receiving Chemotherapy and Radiotherapy

In chemotherapy, patients undergoing infusion may report sensations of pain or a burning feeling at the site of administration. This discomfort can range from mild irritation to severe, debilitating pain, often accompanied by swelling and edema around the infusion site. Visible changes in the skin, such as erythema or redness, may also occur, indicating inflammation and tissue damage. In more severe cases, blistering or the formation of vesicles may be observed, signifying significant injury to the surrounding tissues. Patients may experience necrosis, where skin cells die, leading to the development of ulcers or necrotic lesions [4,5]. Importantly, decreased mobility or impaired function in the affected limb may occur, particularly in instances of substantial swelling or tissue damage [5]. 

In radiotherapy, patients may experience a range of symptoms indicative of extravasation. Skin changes, such as redness, warmth, or localized inflammation, may develop in the treatment area, reflecting radiation-induced tissue damage [10,60]. Patients may report pain, tenderness, or discomfort at the site of irradiation, varying in intensity based on the radiation dose and duration of exposure [10]. Severe cases of extravasation can lead to the formation of skin ulcers or open wounds, indicating significant tissue necrosis and damage. Delayed healing of wounds in the radiation treatment area may prolong the recovery process, with changes in skin texture, such as thickening or scarring, observed over time [61].

Prompt recognition and a response to the signs and symptoms of extravasation can minimize patient morbidity and optimize treatment outcomes. Early intervention, including discontinuation of the infusion, application of cold compresses, and the administration of appropriate antidotes or supportive care measures, can help mitigate the effects of extravasation and prevent long-term complications. Regular assessments and communication with patients regarding any discomfort or changes at the treatment site are essential to ensure the timely intervention and optimal management of extravasation events.

### 6.2. Challenges in Diagnosing Extravasation and Distinguishing It from Other Complications

Diagnosing extravasation, particularly in the context of cancer treatment with chemotherapy and radiotherapy, presents a multifaceted challenge for healthcare providers. The overlapping symptoms and potential complications associated with extravasation make it crucial to differentiate it from other treatment-related reactions or conditions accurately.

One of the primary challenges in diagnosing extravasation is the variability in presenting symptoms [53,62]. While some patients may experience immediate pain, swelling, or erythema at the infusion site, others may exhibit more subtle or delayed signs, complicating the diagnostic process. Moreover, extravasation reactions can manifest differently depending on the type of chemotherapy drugs or radiation techniques used, further complicating diagnosis.

Distinguishing extravasation from other treatment-related reactions, such as infusion reactions or dermatitis, requires a comprehensive assessment of the clinical signs and symptoms [63]. For example, infusion reactions may present with symptoms like fever, chills, or allergic manifestations, which may not be typical of extravasation. Similarly, dermatitis or radiation dermatitis may cause skin changes such as erythema, itching, or peeling, resembling extravasation in some cases.

Furthermore, extravasation must be differentiated from other complications, such as cellulitis, thrombophlebitis, or compartment syndrome, which may present with similar clinical features [11,64]. Cellulitis, characterized by localized inflammation and infection of the skin and subcutaneous tissues, can mimic the erythema and swelling seen in extravasation. Thrombophlebitis, inflammation of the vein with associated pain and tenderness, may occur concurrently with extravasation or independently, complicating diagnosis. Compartment syndrome, characterized by increased pressure within a muscle compartment leading to tissue ischemia, can result from severe extravasation injuries, necessitating prompt recognition and intervention.

Additionally, the timing of symptom onset and progression may provide valuable clues in differentiating extravasation from other complications [23]. Extravasation reactions typically occur during or shortly after infusion, with symptoms worsening over time if left untreated [65]. In contrast, other conditions may present with delayed or persistent symptoms, necessitating careful monitoring and follow-up assessments.

Given the complexity of diagnosing extravasation and distinguishing it from other complications, healthcare providers must rely on a combination of clinical judgment, patient history, and diagnostic tests, such as ultrasound or tissue biopsy, when necessary. Timely recognition and intervention are essential to minimize tissue damage and optimize patient outcomes, highlighting the importance of vigilant monitoring and communication among multidisciplinary healthcare teams involved in cancer care.

### 6.3. Diagnostic Tools and Techniques Used in Confirming Extravasation for Both Treatment Modalities

To confirm extravasation in cancer treatment, whether during chemotherapy or radiotherapy, requires a versatile approach involving clinical assessment, diagnostic imaging, and sometimes tissue sampling. Healthcare providers utilize a variety of diagnostic tools and techniques to accurately identify extravasation events and assess the extent of tissue damage.

In the context of chemotherapy, clinical assessment plays an important role in the initial identification of extravasation [63,66]. Healthcare providers carefully inspect the infusion site for signs of extravasation, including pain, swelling, erythema, and blistering. Patient-reported symptoms, such as discomfort or changes in sensation at the infusion site, also inform the diagnostic process. While clinical evaluation provides valuable insights, additional diagnostic tools may be necessary to confirm extravasation and assess the tissue damage.

Ultrasound imaging is commonly employed to visualize the extent of extravasation and assess tissue involvement [67,68,69]. Ultrasonography enables healthcare providers to identify fluid accumulation, tissue edema, and structural changes in the affected area, aiding in the characterization of extravasation injuries. Doppler ultrasound may also be utilized to assess vascular integrity and blood flow, particularly in cases where vascular compromise is suspected.

In some instances, more invasive diagnostic techniques, such as tissue biopsy, may be warranted to confirm extravasation and evaluate tissue necrosis [70]. Tissue biopsy allows for the histological examination of affected tissues, providing definitive evidence of extravasation and guiding treatment decisions. While less commonly utilized due to its invasive nature, a tissue biopsy may be indicated in cases of diagnostic uncertainty or where there is a suspicion of severe tissue damage.

In radiotherapy, diagnostic tools and techniques for confirming extravasation differ slightly from those used in chemotherapy. Clinical assessment remains paramount, with healthcare providers closely monitoring patients for signs and symptoms of tissue damage in the radiation treatment area. Imaging modalities, including CT and magnetic resonance imaging (MRI), may be utilized to visualize tissue changes and assess the extent of radiation-induced injury [71].

In cases where extravasation is suspected but not definitively confirmed through clinical assessment and imaging, consultation with specialists, such as dermatologists or plastic surgeons, may be beneficial [63]. These specialists can provide expertise in evaluating tissue damage and guiding management strategies, particularly in cases of severe extravasation requiring surgical intervention or advanced wound care.

## 7. Prevention Strategies

### 7.1. Preventive Measures Aimed at Reducing the Risk of Extravasation during Chemotherapy and Radiotherapy

Preventing extravasation during chemotherapy and radiotherapy is a critical aspect of patient care, which is aimed at minimizing tissue damage and potential complications. Healthcare professionals employ a variety of preventive measures to reduce the risk of extravasation, outlined as follows.

Education and Training: Healthcare providers undergo comprehensive training on recognizing, managing, and preventing extravasation incidents. Patients should also be educated about the signs and symptoms of extravasation, emphasizing the importance of immediate reporting [12,72,73,74].

Vein Assessment: Prior to treatment initiation, healthcare professionals conduct a thorough assessment of the patient’s veins to identify suitable access points [68]. For patients with fragile or difficult-to-access veins, alternative access sites such as central venous catheters may be considered [75].

Proper Catheter Placement: Skilled healthcare professionals ensure the proper placement of IV catheters, minimizing the risk of extravasation [76]. Techniques such as ultrasound guidance may be used to verify a catheter’s placement, especially in patients with challenging vascular access.

Use of Vein Visualization Technology: Vein visualization devices are employed to enhance the visualization of peripheral veins [77,78], aiding in accurate catheter insertion and reducing the risk of extravasation.

Regular Monitoring and Assessment: Infusion sites are frequently monitored during chemotherapy or radiotherapy sessions to detect early signs of extravasation [79]. Symptoms such as pain, swelling, redness, or blanching around the infusion site are assessed promptly.

Chemotherapy Agents and Diluents: Healthcare providers select chemotherapy agents and diluents with lower vesicant properties whenever possible to minimize tissue damage in case of extravasation [12,63].

Temperature Regulation: Proper temperature control of the chemotherapy solutions is ensured to minimize the risk of tissue injury if extravasation occurs [80].

Prompt Recognition and Intervention: Healthcare providers are trained to promptly recognize the signs of extravasation and differentiate between irritants and vesicants. Clear protocols are established for the immediate cessation of infusion upon suspicion of extravasation and the initiation of appropriate interventions [12,63].

Extravasation Kits: Healthcare facilities maintain the availability of extravasation kits containing specific antidotes, such as hyaluronidase for certain vesicant agents, to facilitate prompt treatment if extravasation occurs [12,63].

Documentation and Reporting: All incidents of extravasation are documented meticulously, including details such as the type and volume of infusate, site of extravasation, and actions taken. This information is essential for quality improvement and risk management purposes [63,81].

### 7.2. Importance of Proper Vascular Access Device Selection, Administration Techniques, and Patient Education for Both Modalities

Proper vascular access device selection, administration techniques, and patient education are crucial aspects of ensuring the safe and effective delivery of chemotherapy and radiotherapy. These aspects are delineated as follows.

Vascular Access Device Selection: Choosing the appropriate vascular access device is essential to minimize the risk of extravasation and ensure optimal treatment outcomes. For patients undergoing chemotherapy or radiotherapy, the selection of the vascular access device depends on various factors such as treatment duration, frequency, and the patient’s vascular status. Peripheral venous catheters are commonly used for short-term treatments, while central venous catheters, including peripherally inserted central catheters and implanted ports, are preferred for long-term therapy or when peripheral access is challenging [75,76,82,83]. Proper selection reduces the risk of complications such as thrombosis, infection, and extravasation.

Administration Techniques: Skilled administration techniques are essential to prevent extravasation and ensure the accurate delivery of chemotherapy and radiotherapy. Healthcare professionals must receive adequate training in catheter insertion and maintenance to minimize the risk of complications. Techniques such as ultrasound-guided catheter insertion can improve success rates and reduce the risk of vessel injury [84]. Additionally, proper flushing and locking protocols help maintain catheter patency and reduce the risk of occlusion and infection [85]. Regular assessment of the catheter site and monitoring for signs of complications during treatment sessions are essential components of safe administration techniques.

Patient Education: Educating patients about vascular access devices, administration procedures, and potential complications is vital for their active involvement in their care and treatment adherence [86]. Patients should be informed about the purpose of the vascular access device, its maintenance requirements, and signs of complications such as infection or thrombosis. Patient education also includes instructions on recognizing the early signs of extravasation and the importance of timely reporting to healthcare professionals. Moreover, patients with central venous catheters need to understand the proper care and maintenance of their device to prevent complications and ensure its longevity [87].

### 7.3. Management of Extravasation of Vesicants

Managing the extravasation of vesicants requires prompt and effective action to minimize tissue damage and potential complications. Table 1 categorizes the common vesicant chemotherapy drugs by their types [88,89,90]. 

Several professional organizations and expert groups have developed guidelines for the management of extravasation, particularly in cancer patients who often receive vesicant and irritant chemotherapy agents [4,13,89,90,91]. While the guidelines may vary slightly between organizations, they generally share core principles and steps for management.

A typical management of vesicant extravasation on the limb is described as follows.

A Vesicant Extravasation Management Algorithm

- Stop the infusion: Immediately stop the infusion and leave the IV cannula or catheter in place.
- Aspirate the vesicant: Use a syringe to gently aspirate as much of the extravasated drug as possible through the existing IV cannula.
- Disconnect IV tubing: Disconnect the IV tubing while keeping the needle or cannula in place.
- Mark the area: Gently outline the affected area with a skin marker to monitor the changes.
- Apply appropriate compress: Refer to Table 2 [92,93,94,95,96,97,98,99] for the type of compress (cold or warm) specific to the vesicant involved. Apply the compress intermittently (e.g., 15–20 min every hour) for the first 24 to 48 h.
- Administer the antidote if available: Refer to Table 2 for the specific antidote and administer it according to the guidelines.
- Elevate the affected limb: Elevate the affected limb to reduce swelling and promote reabsorption.
- Pain management: Administer the appropriate analgesics for pain relief.
- Monitor and document: Regularly assess the affected area for signs of improvement or worsening. Document all observations, interventions, and patient responses in the medical record.
- Follow-up care: Consult a specialist if significant tissue damage or necrosis is suspected. Educate the patient on the signs of infection or worsening condition and when to seek further medical attention. Schedule follow-up visits to monitor healing and manage complications.
- Review and improve practice: Conduct a review of the incident to identify areas for improvement. Provide additional training for staff on the proper administration of vesicants and the management of extravasation.

**Figure 7:**
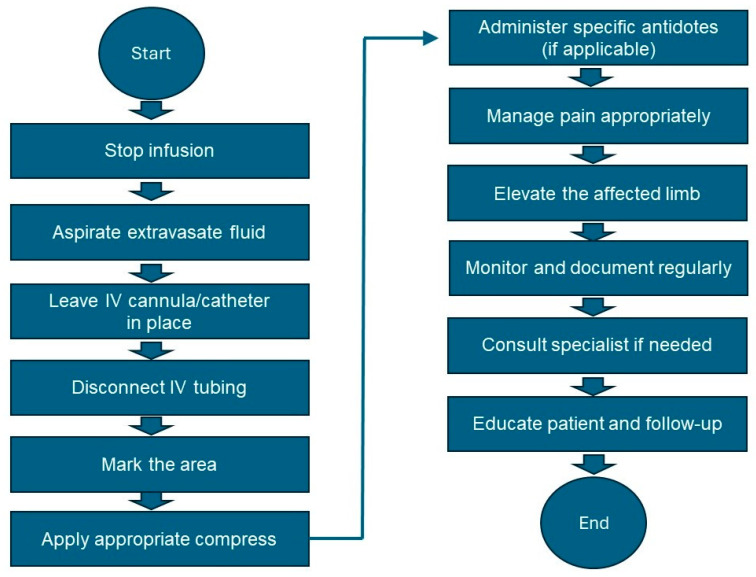
Key steps of a management plan for extravasation of vesicants on an affected limb.

The above algorithm is graphically outlined in Figure 7.

## 8. Complications and Long-Term Effects

Extravasation during chemotherapy or radiotherapy administration can lead to a spectrum of complications [5,11,100], varying from mild discomfort to severe tissue damage and long-term sequelae. Immediate tissue damage is often evident at the infiltration site, manifesting as pain, swelling, redness, and, in severe cases, tissue necrosis. This damage can extend to nerves, resulting in neurotoxicity symptoms like numbness, tingling, weakness, or neuropathic pain, which may become permanent in severe cases. Moreover, muscles and joints may be affected, leading to stiffness, a limited range of motion, and impaired mobility, potentially resulting in chronic pain and functional impairment if not promptly addressed.

Severe extravasation injuries can also cause scarring and fibrosis of the affected tissues [11,101,102], leading to long-term cosmetic deformities and functional limitations. A breakdown of tissue integrity following extravasation increases the risk of secondary infection, leading to cellulitis, abscess formation, or systemic infection if untreated. Additionally, irritation of blood vessels by extravasated agents can trigger thrombus formation [63,103], resulting in local thrombophlebitis or thromboembolic events like deep vein thrombosis or pulmonary embolism.

Moreover, the suboptimal delivery of chemotherapy or radiotherapy agents to the intended target site due to extravasation can compromise treatment efficacy and impact patient outcomes [104]. Extravasation events can also cause distress and anxiety for patients, affecting their emotional well-being and quality of life [105]. Fear of recurrence or future complications may further impact treatment adherence.

In severe cases, extravasation injuries may lead to long-term disability, requiring extensive medical intervention, rehabilitation, and supportive care. Functional impairment and chronic pain may persist, significantly affecting the patient’s quality of life. Additionally, extravasation events may result in legal and ethical dilemmas, particularly if complications arise due to negligence or inadequate management [106]. Healthcare providers must adhere to the professional standards of care, ensuring comprehensive documentation and reporting of extravasation incidents.

## 9. Radiopharmaceutical Extravasation

Radiopharmaceutical therapy (RPT) is emerging as a safe and effective targeted approach to treating many types of cancer [107,108,109]. This innovative treatment involves delivering radiation directly to cancer cells using specially designed pharmaceuticals. These pharmaceuticals either bind specifically to cancer cells or accumulate in them through natural physiological processes. The radionuclides used in RPT emit photons, which can be captured through imaging techniques, allowing healthcare providers to non-invasively track where the therapeutic agents are distributed in the body.

Compared to most other systemic cancer treatments, RPT has demonstrated significant effectiveness with minimal side effects [107]. Traditional treatments, such as chemotherapy and radiation therapy, often affect healthy cells and can cause severe side effects. In contrast, RPT targets cancer cells more precisely, reducing damage to healthy tissue and resulting in fewer and milder side effects.

The U.S. Food and Drug Administration’s approval of several RPT agents highlights the growing recognition of this treatment’s potential [107]. These approvals mark a significant advancement in cancer therapy, providing new options for patients who may not have responded well to conventional treatments. As research continues, it is expected that more RPT agents will be developed and approved, further expanding the range of cancers that can be treated with this approach.

In addition to its clinical benefits, RPT offers practical advantages—the ability to image the distribution of radiopharmaceuticals in real time means that healthcare providers can monitor the treatment’s progress and make adjustments as needed, leading to more personalized and effective care. This precision medicine approach is at the forefront of modern cancer treatment, aligning with the broader movement towards treatments tailored to individual patients’ needs.

However, radiopharmaceutical extravasation is a significant concern in nuclear medicine and radiotherapy, where radiopharmaceuticals are used for both diagnostic imaging and therapeutic purposes [10,110,111]. This phenomenon occurs when a radiopharmaceutical leaks from the vein into the surrounding tissues during IV administration. Understanding the implications, mechanisms, and management strategies of radiopharmaceutical extravasation is crucial for optimizing a patient’s care and outcomes.

Radiopharmaceutical extravasation can lead to several severe local and systemic complications [10]. Local tissue damage is one of the most immediate concerns. The radioactive nature of radiopharmaceuticals can cause significant radiation-induced damage to the surrounding tissues at the site of extravasation, resulting in pain, swelling, erythema, ulceration, and even necrosis. Additionally, in therapeutic settings, extravasation can lead to inappropriate dosimetry [10,112,113]. The intended therapeutic dose may not reach the target tissue, reducing treatment efficacy and potentially necessitating additional treatments. In diagnostic imaging, extravasation can lead to poor image quality and diagnostic inaccuracies. The radiopharmaceutical may not distribute properly within the body, leading to false-negative or false-positive results [114,115], complicating the diagnostic process and potentially delaying appropriate treatment.

Radiopharmaceutical extravasation typically occurs due to improper injection technique, vascular fragility, and high injection pressure [10,116]. Incorrect needle or catheter placement can lead to leakage into the surrounding tissue. Patients with fragile veins, often due to age, chemotherapy, or other underlying conditions, are at higher risk of extravasation. Additionally, rapid injection or the use of high pressure can cause rupture of the vessel wall, leading to extravasation.

Effective management of radiopharmaceutical extravasation involves both preventive measures and prompt response strategies [10,111]. Prevention includes ensuring correct needle or catheter placement through the use of ultrasound guidance and experienced personnel, evaluating patient-specific risk factors such as vein integrity and history of previous extravasation, and administering the radiopharmaceutical slowly to reduce the risk of vessel rupture. Management involves promptly stopping the injection upon suspicion of extravasation and removing the needle or catheter, applying cold or warm compresses, depending on the specific radiopharmaceutical, to reduce tissue damage and alleviate symptoms, and closely monitoring the affected area for signs of severe damage while providing supportive care as needed, which may include pain management and wound care.

## 10. Future Directions and Research Opportunities

### 10.1. Gaps in Current Knowledge and Areas for Future Research in Extravasation Prevention and Management for Both Chemotherapy and Radiotherapy

While significant progress has been made in extravasation prevention and management for both chemotherapy and radiotherapy, there are still several gaps in the current knowledge and areas for future research, which are outlined as follows.

- Risk Factors and Predictive Models: Further research is needed to identify additional risk factors for extravasation, including patient-specific factors such as age, comorbidities, and vascular status. Developing predictive models that incorporate these factors could help stratify patients based on their risk of extravasation and guide personalized prevention strategies.
- Novel Prevention Strategies: Current prevention strategies primarily focus on proper vascular access device selection and administration techniques. Future research could explore the efficacy of novel preventive interventions, such as vein mapping technologies, protective dressings, or pharmacological agents, in reducing the incidence of extravasation.
- Early Detection Methods: Research into non-invasive or minimally invasive methods for the early detection of extravasation is warranted. Developing innovative imaging techniques or biomarkers that can accurately detect extravasation at its earliest stages could facilitate timely intervention and prevent tissue damage.
- Optimal Management Protocols: There is a need for standardized management protocols for extravasation events, including clear guidelines on the selection and administration of antidotes, wound care techniques, and follow-up strategies. Comparative studies evaluating the effectiveness of different management approaches and interventions are essential for establishing evidence-based protocols.
- Patient Education and Support: Research focusing on the effectiveness of patient education programs and supportive interventions in enhancing patient awareness, self-management, and coping strategies following extravasation events is needed. Understanding patients’ perspectives, experiences, and information needs can inform the development of tailored educational resources and support services.
- Long-Term Outcomes and Quality of Life: Limited research has examined the long-term consequences of extravasation on patients’ quality of life, functional outcomes, and psychological well-being. Longitudinal studies investigating the impact of extravasation-related complications, such as scarring, neuropathy, or chronic pain, on patients’ long-term health outcomes are necessary.
- Healthcare Provider Training and Competency: Research into effective strategies for training healthcare providers in extravasation prevention, recognition, and management is essential. Assessing the impact of educational interventions, simulation training, and competency assessments on healthcare providers’ knowledge, skills, and confidence in managing extravasation events can help improve patient safety and outcomes.
- Health Economics and Resource Utilization: Evaluating the economic burden of extravasation-related complications, including healthcare resource utilization, hospitalization costs, and productivity losses, is crucial for healthcare decision-making and resource allocation. Cost-effectiveness analyses of different prevention and management strategies can inform policy and practice guidelines.

### 10.2. Emerging Technologies or Therapies That May Improve Extravasation Prevention and Management in Both Treatment Modalities

Emerging technologies and therapies hold great promise for improving extravasation prevention and management in both chemotherapy and radiotherapy. These advancements encompass a wide range of approaches, including innovative medical devices, targeted therapies, and artificial intelligence (AI) applications for image analysis. These promising technologies are outlined as follows.

- Vein Visualization Technologies: Advanced vein visualization devices, such as near-infrared imaging and augmented reality systems, offer real-time visualization of the peripheral veins, enhancing the accuracy of vascular access device placement and reducing the risk of extravasation. These technologies provide healthcare providers with improved guidance for catheter insertion, especially in patients with difficult-to-access veins.
- Smart Catheters and Infusion Systems: The development of smart catheters and infusion systems equipped with sensors and feedback mechanisms enables the real-time monitoring of infusion parameters, including flow rates, pressure, and drug compatibility. These systems can alert healthcare providers to potential extravasation events and automatically adjust the infusion parameters to minimize the risk of tissue damage.
- Targeted Drug Delivery Systems: Targeted drug delivery systems, such as liposomes, nanoparticles, or drug-eluting implants, offer a localized and controlled release of chemotherapy or radiotherapy agents, reducing systemic toxicity and the risk of extravasation-related complications. These technologies allow for the precise delivery of therapeutic agents to tumor tissues while minimizing exposure to healthy tissues.
- Topical Treatments and Wound Care Products: Novel topical treatments and wound care products specifically designed for extravasation injuries offer promising avenues for improving tissue healing and minimizing scarring—advanced wound dressings incorporating growth factors, antimicrobial agents, or tissue-engineered scaffolds to promote tissue regeneration and accelerate wound closure are some of these avenues.
- AI for Image Analysis: AI-based image analysis algorithms can enhance the detection and diagnosis of extravasation events in imaging studies, such as ultrasound, MRI, and CT. These algorithms can automatically identify subtle signs of extravasation, assist healthcare providers in interpreting the imaging findings, and facilitate a timely intervention. This suggestion is subsequently extended as a separate discussion.

## 11. AI for Image Analysis in Extravasation Detection

Detecting extravasation on medical images poses several challenges, particularly when dealing with small abnormal objects or subtle changes in tissue appearance. Conventional image analysis techniques may struggle to accurately identify these abnormalities among complex anatomical structures and imaging artifacts. Moreover, the subjective interpretation of imaging findings by healthcare professionals can introduce variability and may lead to delays in diagnosis and intervention. 

In oncology, there is a growing emphasis on precision medicine, tailoring treatments to each patient’s and tumor’s specific characteristics [117]. This shift enhances personalized chemotherapy and radiotherapy, while advancements in imaging technologies offer improved tissue characterization and response evaluation [104]. However, oncology treatments can lead to both local and systemic changes, underscoring the need for accurate post-treatment imaging interpretation [118,119]. AI shows great promise in rapidly analyzing vast amounts of imaging data, identifying subtle patterns, aiding in early detection, precise tumor characterization, and predicting the treatment response [120,121,122]. Moreover, AI can integrate imaging data with clinical information, enabling personalized treatment strategies and ultimately enhancing patient outcomes [123,124]. Thus, AI holds significant potential in advancing oncology imaging, potentially enabling personalized chemotherapy and radiotherapy.

AI algorithms, especially those based on deep learning architectures like convolutional neural networks (CNNs) [125], hold promise in identifying subtle deviations in tissue appearance, such as faint discolorations or irregular contrast patterns, indicative of extravasation. By training CNNs on annotated datasets of images depicting extravasation events of varying sizes and characteristics, researchers can develop models capable of accurately detecting small abnormal objects with high precision. By automating this detection process, AI can significantly enhance the efficiency and precision of extravasation diagnosis.

The utilization of AI in extravasation detection represents a new frontier in this domain. Advancements in AI algorithms, particularly in the domain of small object detection and segmentation, hold promise for heightened accuracy and sensitivity in identifying extravasation events. Moreover, integrating AI with three-dimensional reconstruction could unlock new dimensions for comprehensive extravasation assessment and characterization.

AI-driven image analysis not only flags potential extravasation events but also offers invaluable assistance to healthcare providers in interpreting imaging findings. By highlighting regions of interest and providing quantitative assessments of extravasation severity and extent, these algorithms can empower clinicians to make informed decisions regarding patient care. Leveraging this information, healthcare providers can formulate tailored treatment strategies and initiate timely interventions to mitigate potential complications.

For small and transient object detection, particularly in medical imaging applications like detecting extravasation, pretrained CNN models that have demonstrated effectiveness in possessing high sensitivity to complex patterns and subtle features are preferable. Below are some pretrained CNN models suitable for this task.

- RetinaNet [126]: This CNN is a single-stage object detection model known for its effectiveness in detecting small and transient objects. It addresses the challenge of class imbalance in dense detection tasks by employing a focal loss function. This enables RetinaNet to assign higher weights to hard-to-detect objects, making it particularly suitable for detecting subtle abnormalities like extravasation.
- Mask R-CNN [127]: This model extends Faster R-CNN by adding a mask prediction branch, enabling pixel-level segmentation in addition to object detection. This makes it well-suited for tasks requiring precise localization of small and transient objects. Mask R-CNN has been successfully applied to various medical imaging tasks and can be adapted for detecting extravasation with high accuracy.
- YOLOv4 [128]: It is an advanced version of the YOLO (you only look once) object detection algorithm known for its speed and accuracy. It utilizes a single neural network to predict bounding boxes and class probabilities for small and transient objects in real time. YOLOv4’s efficiency and effectiveness make it suitable for detecting extravasation events in medical images.
- EfficientDet [129]: This pretrained CNN is a family of efficient and accurate object detection models based on the EfficientNet backbone architecture. These models achieve state-of-the-art performance with significantly fewer parameters compared to traditional models, making them well-suited for resource-constrained environments like medical imaging applications. EfficientDet’s lightweight design and high accuracy make it suitable for detecting small and transient objects like extravasation.
- CenterNet [130]: This net is a simple and efficient object detection model that directly predicts object centers and regresses bounding boxes from them. This approach makes CenterNet particularly effective for detecting small and transient objects with high accuracy. CenterNet’s simplicity and effectiveness make it a viable option for detecting extravasation events in medical images.

These pretrained CNN models provide a strong foundation for detecting small and transient objects like extravasation in medical imaging. Fine-tuning these models on annotated datasets of medical images containing extravasation cases can further enhance their performance and adaptability to specific clinical applications.

## 12. Conclusions

This review emphasizes the critical importance of proactive management and prevention strategies in minimizing the impact of extravasation on cancer patients undergoing both chemotherapy and radiotherapy. Extravasation events can lead to a spectrum of complications, ranging from local tissue damage to long-term disability, significantly affecting patients’ quality of life and treatment outcomes. Thus, prompt recognition, appropriate intervention, and comprehensive follow-up are essential to mitigate the potential adverse effects of extravasation.

To achieve optimal extravasation care, healthcare professionals must prioritize prevention and management efforts as part of comprehensive oncology care. This includes implementing standardized protocols and guidelines, providing comprehensive training and education, fostering multidisciplinary collaboration, investing in research and development of innovative technologies and therapies, and enhancing patient education and support services.

Recommendations for healthcare professionals and policymakers also include advocating for policy initiatives aimed at improving extravasation care standards and promoting patient safety. By integrating AI technologies and proactive management strategies into clinical practice, healthcare providers can effectively mitigate the impact of extravasation on cancer patients, ultimately improving the treatment outcomes and enhancing a patient’s quality of life.

## Figures and Tables

**Table 1:** Common vesicant chemotherapy drugs categorized by their types.

Category	Example Drugs
Anthracyclines	Doxorubicin (Adriamycin)
	Daunorubicin (Cerubidine)
	Epirubicin (Ellence)
	Idarubicin (Idamycin)
Vinca alkaloids	Vincristine (Oncovin)
	Vinblastine (Velban)
	Vinorelbine (Navelbine)
Taxanes	Paclitaxel (Taxol)
	Docetaxel (Taxotere)
Nitrogen mustards	Mechlorethamine (Mustargen)
Other vesicants	Mitomycin-C
	Dactinomycin (Actinomycin D)
	Etoposide (at high concentrations)

**Table 2:** Specific antidotes and compress types.

Vesicant Category	Example Drugs	Compress Type	Specific Antidote	Additional Notes
Anthracyclines	Doxorubicin, Daunorubicin	Cold	Dexrazoxane	Administer dexrazoxane IV within 6 h
Vinca alkaloids	Vincristine, Vinblastine	Warm	Hyaluronidase	Inject hyaluronidase subcutaneously
Taxanes	Paclitaxel, Docetaxel	Cold	None	Follow general extravasation protocols
Nitrogen mustards	Mechlorethamine	Cold	Sodium Thiosulfate	Inject sodium thiosulfate subcutaneously
Other vesicants	Mitomycin-C, Dactinomycin	Cold	None	Follow general extravasation protocols
